# Keggin Heteropolyacid Immobilized on Nanosilica as a Heterogeneous Catalyst for Sugar Dehydration in an Aqueous Medium

**DOI:** 10.3390/molecules30204097

**Published:** 2025-10-15

**Authors:** Vincenzo Campisciano, Serena Lima, Giuseppe Marcì, Filippo Vitale, Maria Luisa Saladino, Francesco Giacalone, Elisa I. García-López

**Affiliations:** 1 Department of Biological, Chemical and Pharmaceutical Sciences and Technologies (STEBICEF) and INSTM UdR–Palermo, University of Palermo, Viale delle Scienze, Ed. 17, 90128 Palermo, Italy; vincenzo.campisciano@unipa.it (V.C.); marialuisa.saladino@unipa.it (M.L.S.); francesco.giacalone@unipa.it (F.G.); elisaisabel.garcialopez@unipa.it (E.I.G.-L.); 2 Dipartimento di Ingegneria, Università di Palermo, Viale delle Scienze, Ed. 6, 90128 Palermo, Italy; serena.lima@unipa.it; 3 Consiglio Nazionale delle Ricerche, Istituto per i Processi Chimico Fisici, Viale Ferdinando Stagno d’Alcontres 37, 98158 Messina, Italy

**Keywords:** biomass, keggin heteropolyacid, nanosilica, sugars dehydration

## Abstract

The dehydration of fructose and glucose to 5-hydroxymethylfurfural (HMF) in water solution was carried out in the presence of functionalized heteropolyanion-based heterogeneous catalysts. Two catalysts were prepared by immobilizing the Keggin polyoxometalate H_3_PW_12_O_40_ (PW_12_) onto nanoSiO_2_ by the use of imidazoline and -SO_3_^−^ surface species through acid–base reactions. The catalysts were characterized by N_2_ adsorption–desorption isotherms, XRD, TGA, FTIR, solid-state NMR, SEM, and acidity–basicity measurements. Catalytic reactions in batch conditions were performed at 165 °C in the presence of suspended catalysts, and the yield of furfural and 5-hydroxymethylfurfural (5-HMF) was determined. The catalytic activity of the materials was tested for sugars at 1M concentration in a water solution. The valorization of sugars (fructose and glucose) was found to be more effective in the case of fructose. Furthermore, the two catalysts in which the heteropolyacid was immobilized showed activity similar to that observed for naked PW_12_ (reaction in homogeneous phase), despite the heterogeneous nature of the process, but with the advantage of easier separation at the end of the reaction by simple filtration. The material’s substantial stability was demonstrated through three consecutive catalytic test cycles, in which the same catalyst was recovered after each experiment and washed several times with hot water. Finally, tests devoted to the valorization of sugars contained in wastewater from the brewing industry provided a poor yield in 5-HMF, indicating that the catalysts prepared here were, unfortunately, not suitable for this transformation under the conditions tested. This was because the catalysts prepared in this work showed a low capacity to transform glucose (the most present sugar in the carbohydrate fraction of this biomass) into furans.

## 1. Introduction

Vegetable biomass, constituted of polysaccharides, hemicellulose, and cellulose, is a sustainable source of renewable carbon. The chemical composition of biomass possesses a huge potential as a source of low-molecular-weight compounds. Biomass is a green, easily available, renewable source suitable for obtaining fuels or chemicals [1]. The valorization of hexoses, the most abundant monosaccharides derived from biomass, represents a relevant scientific issue worth exploring. The dehydration reaction of sugars to produce furans, including 5-hydroxymethylfurfural (5-HMF) and furfural, is an important approach to valorize biomass into useful chemicals. 5-HMF is considered a platform chemical [2], i.e., a starting substance used to obtain, for instance, fuels such as 2,5-dimethylfuran (DMF) [3] or polyester building block chemicals [4].

Also, furfural represents a useful resource for the obtaining of other valuable products, such as furoic acid, furfuryl alcohol, furan, tetrahydrofuran, 2-methyltetrahydrofuran, and related resins. In addition, insoluble humins can be formed by condensation reaction starting from furans, whereas levulinic and formic acid are obtained by hydration of 5-HMF.

**Figure 1:**
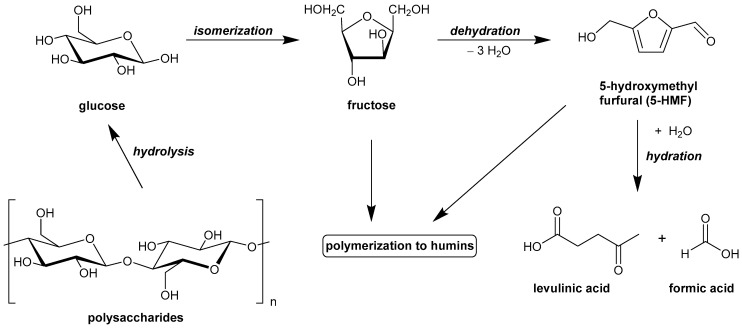
Scheme of the reaction for 5-HMF and furfural production from the sugars provided by the biomass.

Usually, dehydration reactions are carried out in a homogeneous phase with the aid of mineral acids. However, the use of a solid catalyst could offer a more sustainable alternative in light of easier catalyst recovery and limited release into the environment. If, on the one hand, 5-HMF can be obtained by the direct dehydration of fructose in the presence of catalytic amounts of an acidic material possessing Brønsted sites (as well as in the presence of a mineral acid), on the other hand, the conversion of glucose into 5-HMF requires an additional preliminary step of isomerization to fructose, as shown in Figure 1. Specifically, the first step in the Brønsted acid-mediated conversion of glucose to 5-HMF, namely the isomerization of glucose to fructose, can be catalyzed by enzymes or Lewis acids [5,6]. Therefore, the development of an efficient and direct industrial conversion of glucose into 5-HMF would represent the most beneficial, economical, and environmentally feasible route. The selectivity of the conversion of glucose into 5-HMF can be decreased by several side reactions. These reactions are depicted in Figure 1 and include the following: (i) cross-condensation of glucose/fructose with other reaction products; (ii) self-condensation of 5-HMF with the formation of slightly soluble or insoluble oligomers/polymers (humins); (iii) rehydration of 5-HMF with the formation of levulinic and formic acids.

The valorization of biomass containing polysaccharides to obtain higher-value products, such as furans, is an important area of research with significant implications for sustainable energy production and environmental impact. Biomass matter contains polysaccharides, which can give rise to monomeric sugars, such as glucose, xylose, and others, that can be exploited to yield high-value products. Polymeric sugars in biomass waste can be extracted and hydrolyzed to simple sugars by means of a chemical or physical treatment and further dehydrated to yield as main products furfural [7] and/or 5-hydroxymethyl-furfural (5-HMF) [8,9].

Many acid catalysts have been used to transform carbohydrates into 5-HMF, including mineral acids [10], metal ions in solution [11], ion-exchange resins [4], supported heteropoly acids (HPAs) [12], Nb_2_O_5_ [8], and Niobium phosphate [9], among other heterogeneous acidic catalysts. Moreover, a variety of solvents, including water, organic solvents, ionic liquids (ILs), and several biphasic water/organic systems, have been involved [13]. The use of ionic liquids for the conversion of carbohydrates to 5-HMF has been extensively reported and thoroughly reviewed [14].

This work reports the preparation and characterization of two catalysts in which the Keggin H_3_PW_12_O_40_ has been immobilized onto two functionalized nanoSiO_2_. The prepared catalysts were then used as heterogeneous catalysts for the dehydration of fructose or glucose (to form furans) contained in synthetic aqueous solutions or in sugars contained in wastewater derived from the brewing industry, resulting in recyclability in the former case.

## 2. Results and Discussion

### 2.1. Characterization of the Catalysts

**Scheme 1:**
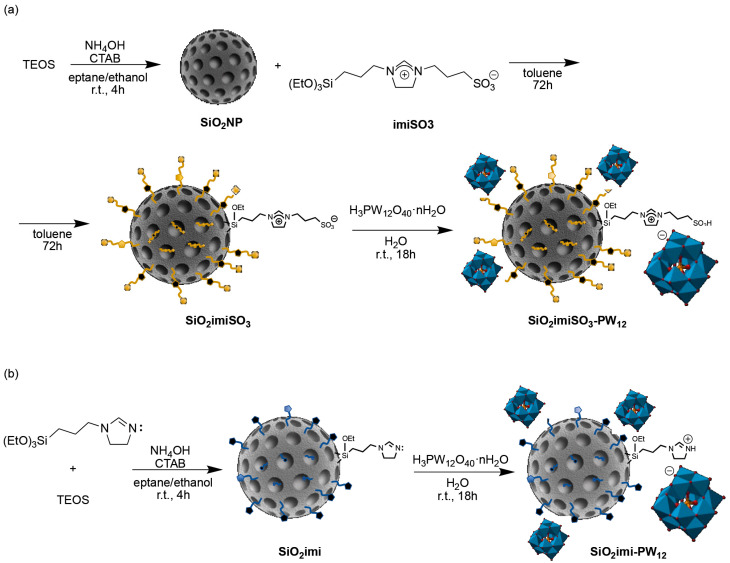
Procedures adopted for the preparation of supported heteropolyacid (**a**) **SiO_2_imiSO_3_-PW_12_** and (**b**) **SiO_2_imi-PW_12_**.

As mentioned in the Experiment section, the two catalysts based on nanosilica were prepared using different strategies. In the first case, **SiO_2_NP** was prepared through an oil-in-water emulsion protocol, followed by the grafting of **imiSO_3_** in refluxing toluene. Then, the heteropolyacid H_3_PW_12_O_40_ was immobilized via an acid–base reaction, allowing the achievement of **SiO_2_imiSO_3_-PW_12_** (Scheme 1a). On the other hand, catalyst **SiO_2_imi-PW_12_** was prepared by starting from **SiO_2_imi** nanoparticles, which were obtained by emulsion co-condensation of triethoxy-3-(2-imidazolin-1-yl)propylsilane and TEOS, and then reacted with heteropolyacid H_3_PW_12_O_40_ in water at room temperature (Scheme 1b).

**Figure 2:**
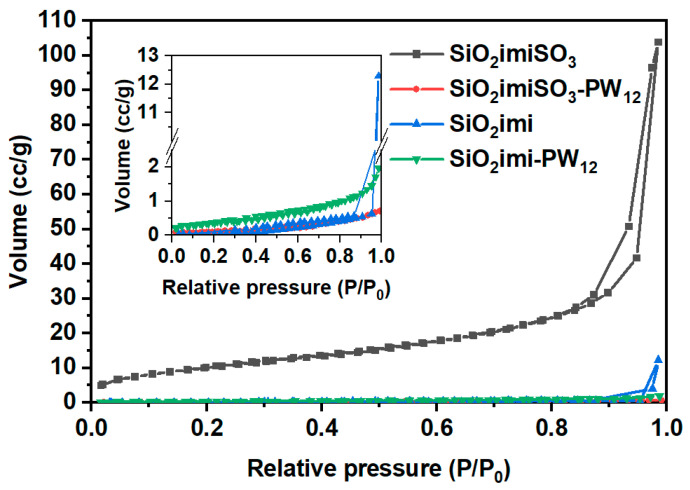
N_2_ adsorption/desorption isotherms of catalysts and their respective supports. The inset reports the enlargement of the N_2_ adsorption and desorption isotherm curves recorded for SiO_2_imiSO_3_-PW_12_, SiO_2_imi, and SiO_2_imi-PW_12_ (see Figures S1–S4 for magnification and relative pore distribution curves).

In order to study the textural properties of the prepared materials, N_2_ adsorption and desorption isotherms were recorded, and they are shown in Figure 2. From the study of these isotherms, it was possible to obtain some of the structural properties of the samples, which are listed in Table 1.

The specific surface area (S_BET_), determined using the standard BET method, is 37 m^2^∙^−1^ for **SiO_2_imiSO_3_** and 3.2 m^2^∙g^−1^ for **SiO_2_imiSO_3_-PW_12_**. This significant reduction in S_BET_ is attributed to the immobilization of the heteropolyacid on the superficial functionalities, including those within the pore network.

The pore filling likely restricts access to smaller pores, leaving larger pores underutilized, which results in a decreased cumulative pore volume and slightly increased w_BJH_. In contrast, **SiO_2_imi** and **SiO_2_imi-PW_12_** exhibit an opposite trend. Catalyst immobilization increases S_BET_ from 3.9 to 15 m^2^∙g^−1^, indicating that the catalytic moieties are effectively anchored, potentially generating new accessible surface areas [15]. While the cumulative pore volume and size slightly decrease, this is consistent with partial pore filling. These variations can likely be ascribed to differences in the siliceous framework, as the material is either (*i*) functionalized post-synthesis (i.e., **SiO_2_imiSO_3_**) or (*ii*) synthesized through a co-condensation route (i.e., **SiO_2_imi**).

**Figure 3:**
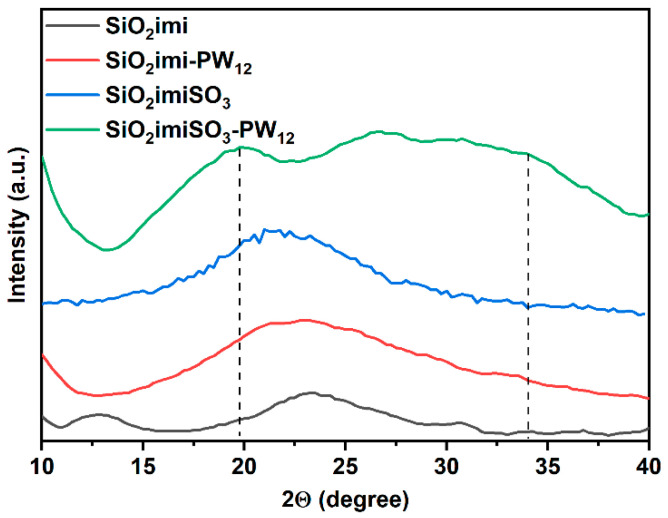
XRD patterns of the functionalized materials and the corresponding PW_12_-supported catalyst.

In order to investigate the structure of the prepared materials, XRD patterns were recorded. The XRD patterns of POM functionalized materials and those of the supporting precursors are shown in Figure 3. As expected, both of the supporting materials (e.g., **SiO_2_imi** and **SiO_2_imiSO_3_**) displayed broad bands centered at approximately 23°, typically ascribed to amorphous silica-based materials, indicating that the functionalization did not alter the structure of the nanoparticles. The immobilization of the Keggin structure on **SiO_2_imi** did not modify the amorphous nature of the material, while **SiO_2_imiSO_3_-PW_12_** features the characteristic peaks of the H_3_PW_12_O_40_ at 19.8° and 34°, which broaden because of the well-dispersion and the confinement within the pore of the catalyst [16].

**Figure 4:**
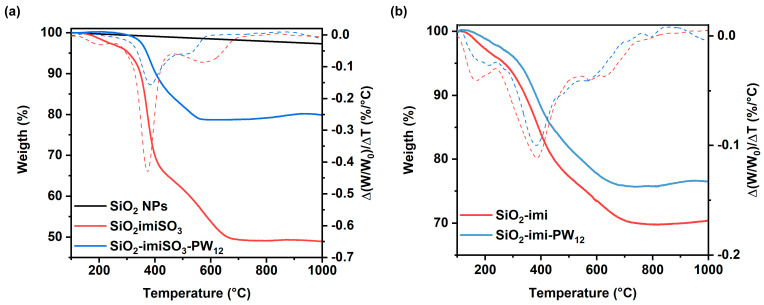
Thermogravimetric analysis (solid lines) and differential thermogravimetric analysis (dashed lines) of (**a**) **SiO_2_NP**, **SiO_2_imiSO_3_** and **SiO_2_imiSO_3_-PW_12_** and (**b**) **SiO_2_imi** and **SiO_2_imi-PW_12_** recorded in air flow.

Keggin heteropolyacid H_3_PW_12_O_40_ and all the prepared materials were, moreover, subjected to thermogravimetric analysis under air flow, as shown in Figure 4. **SiO_2_NP** exhibits a high thermal stability, displaying weight loss at 800 °C of less than 1 wt%, as evidenced in Figure 4a (black line). Interestingly, the reaction with **imiSO_3_** led to a highly functionalized hybrid material, with organic loading in **SiO_2_imiSO_3_** of about 51% (weight loss of ca. 51 wt%, as evidenced by the red line in Figure 4a), corresponding to 1.98 mmol∙g^−1^.

Hence, the subsequent immobilization of the heteropolyacid in **SiO_2_imiSO_3_-PW_12_** resulted in an increase in the inorganic residue (from 49 to 79%), providing catalytic loading of 0.66 mmol g^−1^ of H^+^ (blue line in Figure 4a). For the material obtained through the co-condensation method **SiO_2_imi** (dashed red line in Figure 4b), albeit possessing a higher organic loading (2.67 mmol g^−1^), once treated with the polyoxometalate, the final catalyst showed just a little increase (6.5%), indicating catalytic loading of 0.24 mmol g^−1^ of H^+^ (dashed cyan line in Figure 4b). The differences between the two heterogeneous catalysts are evident here, as the functionalization of the **SiO_2_NP** occurs on the external surface, where organic moieties are present, whereas the nanomaterial obtained via co-condensation has imidazoline moieties involved in generating the mesostructured backbone and subsequently not available for reaction with POM.

FTIR is useful to evaluate the integrity of the heteropolyacid structure in the composites.

The Keggin anion (PW_12_) possesses four types of oxygen, which provide four characteristic transitions in the range 1200–700 cm^−1^. These bands are assigned to the typical vibrations of the Keggin unit at 1070 (stretching vibration (P-Oi)), 970 (stretching vibration of W = O), 890, and 810 cm^−1^ (corner- and edge-sharing W–O–W vibrations) according to the literature data [17,18,19]

**Figure 5:**
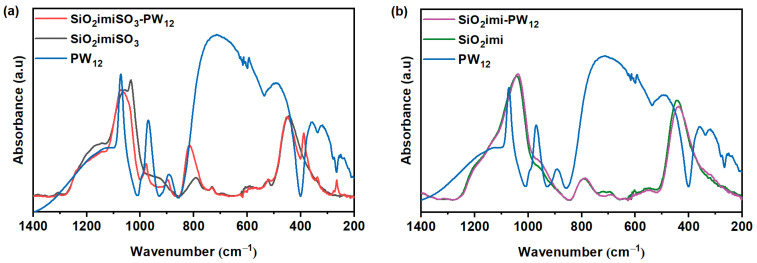
ATR-FTIR spectra of (**a**) **PW_12_**, **SiO_2_imiSO_3_**, and **SiO_2_imiSO_3_-PW_12_**, and (**b**) **PW_12_**, **SiO_2_imi**, and **SiO_2_imiPW_12_**.

Figure 5 presents a comparison between the ATR-FTIR spectra of pristine PW_12_ (blue line) and those of the heteropolyacid immobilized on silicon-based supports, namely SiO_2_imiSO_3_ and SiO_2_imi, respectively. In Figure 5a, the characteristic vibrational bands of the Keggin structure at approximately 1070, 980, and 890 cm^−1^ are observed to undergo a slight blueshift upon immobilization. These spectral changes suggest that both the core Keggin structure, including the corner- and edge-shared W–O–W, is preserved within the hybrid framework.

The shift to higher wavenumbers in the W–O–W stretching vibrations is consistent with strong interactions between the oxygens of the PW_12_ heteropolytungstate anion and imidazolium cation in the prepared nanomaterial, as previously reported [20]. These frequency shifts are indicative of the enhanced stabilization of the heteropolyacid within the material. In addition, interactions between the PW_12_ and hydroxyls of the oxide supports, as reported in the literature for SiO_2_ [21], cannot be excluded. Indeed, according to Rocchiccioli-Deltcheff et al. [22], a shift in the characteristic IR bands of the polyoxometalate can be attributed to various interactions, including hydrogen bonding and hydration effects involving water of crystallization. As a result, the P-O and W=O stretching vibration modes could be broadened or shifted with respect to the bare PW_12_ spectrum due to H-bonding interaction between the oxygen atom of the Keggin anion and hydroxyl groups. At the same time, the near-maintenance of vibrations at 1080 and 890 cm^−1^ allows us to conclude the preservation of the primary structure geometry of the Keggin cluster in the catalysts. A further diagnostic feature is the disappearance of the absorption band at 1027 cm^−1^ in the **SiO_2_ImiSO_3_-PW_12_** hybrid. This band, attributable to the S=O stretching mode of the sulfonic acid group in the betaine-functionalized ionic liquid, is absent after the incorporation of PW_12_. Its suppression suggests the proton transfer and subsequent formation of a strong ion-pair interaction between the heteropolyacid and the sulfonic moiety, reinforcing the conclusion of successful chemical anchoring of PW_12_ to the functionalized silica surface. Conversely, the ATR-FTIR analysis of the hybrid material **SiO_2_Imi-PW_12_** (pink line), displayed in Figure 5b, reveals a significant overlap between the spectrum of the catalyst (pink line) and that of the functionalized support **SiO_2_Imi** (green line). The apparent lack of distinction between the composite and the support may be attributed to several factors. First, the amount of immobilized PW_12_ may be below the detection limit of FTIR in the configuration used, or its characteristic bands may be masked by the signals of the imidazoline-functionalized organosilica matrix. Another possibility could rely on the strong dispersion of the PW_12_ cluster within the matrix or structurally distorted due to strong interactions with the support’s functional groups (e.g., acid–base or hydrogen-bonding interactions).

**Figure 6:**
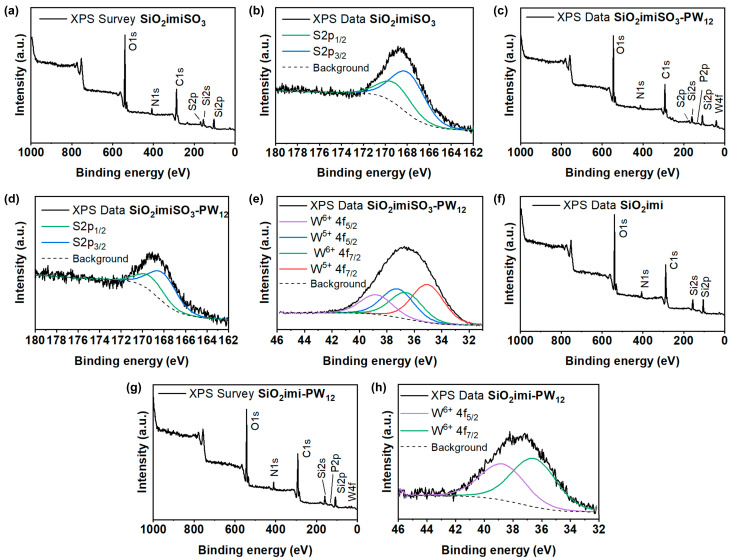
(**a**) XPS survey spectrum, and (**b**) S2p region of SiO_2_imiSO_3_; (**c**) XPS survey spectrum, (**d**) S2p, and (**e**) W4f regions of SiO_2_imiSO_3_-PW_12_; (**f**) XPS survey spectrum of SiO_2_imi; (**g**) XPS survey spectrum, and (**h**) W4f region of SiO_2_imi-PW_12_.

X-ray photoelectron spectroscopy (XPS) of all the prepared materials, before and after the immobilization of PW_12_, allowed for the analysis of their surface elemental composition, as well as the determination of the chemical state of the atoms (Figure 6). Both survey and S2p high-resolution spectra of **SiO_2_imiSO_3_** (Figure 6a,b) are compared with the corresponding spectra of **SiO_2_imiSO_3_-PW_12_** (Figure 6c,d). No remarkable differences were detected with the exception of the presence of phosphorus and tungsten atoms due to the immobilization of PW_12_. Both S2p high-resolution spectra of the analyzed materials display a doublet at 169.8 and 168.4 eV, corresponding to S2p_1/2_ and S2p_3/2_, respectively [23]. The analysis of the W4f high-resolution spectrum of **SiO_2_imiSO_3_-PW_12_** (Figure 6e) shows the presence of four peaks grouped into two doublets at 38.8, 37.3, 36.7, and 35.1 eV, due to the presence of tungsten in the W^6+^ and W^5+^ oxidation state, and assigned to W^6+^ 4f_5/2_, W^5+^ 4f_5/2_, W^6+^ 4f_7/2_, and W^5+^ 4f_7/2_, respectively [20,24].

The W^6+^/W^5+^ atomic ratio was 40:60. The elemental composition of **SiO_2_imi** and **SiO_2_imi-PW_12_** was determined by the acquisition of the XPS survey spectra reported in Figure 6f,g. In addition, the W4f high-resolution spectrum of **SiO_2_imi-PW_12_**, shown in Figure 6h, highlights the presence of only the higher oxidation state of tungsten, namely W^6+^, which gives rise to the doublet at 38.8 and 36.7 eV corresponding to W^6+^ 4f_5/2_ and W^6+^ 4f_7/2_, respectively.

**Figure 7:**
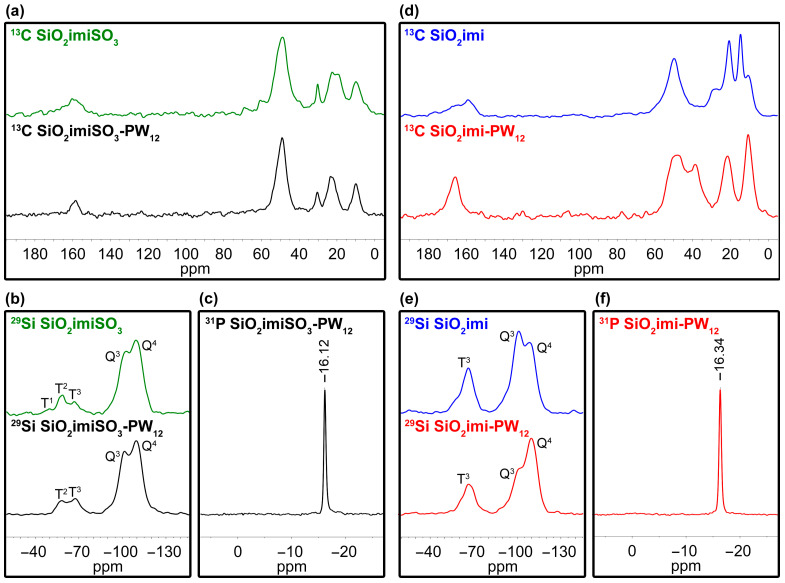
(**a**) ^13^C {^1^H} and (**b**) ^29^Si {^1^H} spectra of SiO_2_imiSO_3_ and SiO_2_imiSO_3_ -PW_12_, and (**c**) ^31^P {^1^H}CP-MAS NMR spectra of SiO_2_imiSO_3_ -PW_12_; (**d**) ^13^C {^1^H} and (**e**) ^29^Si {^1^H} spectra of SiO_2_imi and SiO_2_imi -PW_12_, and (**f**) ^31^P {^1^H}CP-MAS NMR spectra of SiO_2_imi -PW_12_.

All the prepared materials were also characterized by means of solid-state spectroscopy. ^13^C, ^29^Si, and ^31^P {^1^H}CP-MAS spectra are reported in Figure 7. The ^13^C {^1^H}CP-MAS spectrum of **SiO_2_imiSO_3_** confirmed the good outcome of the grafting of the SO_3_^−^ containing silane derivative onto **SiO_2_NP** (Figure 7a, green line). The immobilization of Keggin H_3_PW_12_O_40_ species caused no remarkable changes in the ^13^C solid-state NMR spectrum (compare black and green lines in Figure 7a).

Conversely, ^29^Si {^1^H}CP-MAS spectra showed that the immobilization of H_3_PW_12_O_40_ resulted in a higher degree of condensation of the grafted silane groups with the superficial hydroxyl groups of **SiO_2_NP**. This finding was witnessed by the disappearance of the T^1^ peak at −49.8 ppm, the decrease in intensity of the T^2^ signal at −58.7 ppm, and the consequent raising of the T^3^ signal at −66.8 ppm passing from **SiO_2_imiSO_3_** to **SiO_2_imiSO_3_-PW_12_** (compare green and black lines in Figure 7b). Furthermore, ^29^Si solid-state NMR spectra showed a little increase in the Q^4^/Q^3^ ratio with respect to the **SiO_2_imiSO_3_** precursor material.

The ^31^P {^1^H}CP-MAS spectrum of **SiO_2_imiSO_3_-PW_12_** showed a sharp peak at −16.1 ppm, characteristic of crystalline H_3_PW_12_O_40_, indicating that the native structure of Keggin polyoxometalate was retained (Figure 7c) [25,26].

Unlike the case of **SiO_2_imiSO_3_-PW_12_**, the immobilization of Keggin H_3_PW_12_O_40_ onto **SiO_2_imi** caused some changes in the ^13^C {^1^H}CP-MAS spectrum passing from **SiO_2_imi** to **SiO_2_imi-PW_12_**, especially in the 30–60 ppm region due to the protonation of imidazoline moiety, which affected the chemical shifts at which carbon atoms of methylene groups of imidazoline ring and that belonging to the propyl chain directly bonded to the nitrogen atom resonate (compare blue and red lines in Figure 7d).

Interestingly, the comparison of ^29^Si solid-state NMR spectra of **SiO_2_imi** and **SiO_2_imi-PW_12_** highlighted that all the silane groups of triethoxy-3-(2-imidazolin-1-yl)propylsilane were already fully condensed in the **SiO_2_imi** material, as shown by the presence of the only T^3^ signal at −66.3 ppm. However, the immobilization of H_3_PW_12_O_40_ species caused a marked increase in the Q^4^/Q^3^ ratio with respect to the **SiO_2_imi** precursor material, which is ascribed to further condensation of free silanol groups present in the bulk structure of silica-based hybrid material (Figure 7e, blue and red lines).

As in the case of **SiO_2_imiSO_3_-PW_12_**, the ^31^P {^1^H}CP-MAS spectrum of **SiO_2_imi-PW_12_** also showed a sharp peak at −16.3 ppm that confirmed the structural integrity of the Keggin polyoxometalate (Figure 7f).

**Figure 8:**
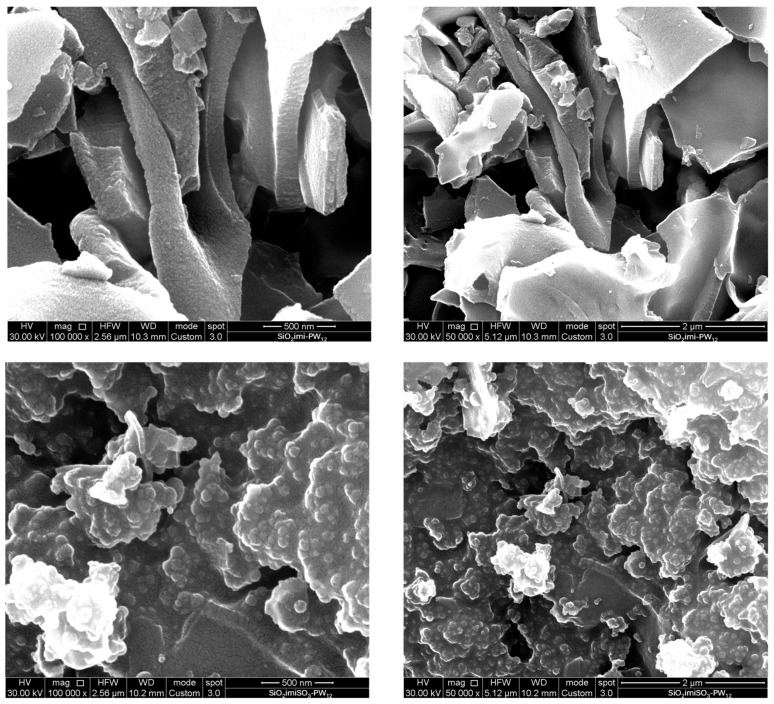
SEM images of **SiO_2_imi-PW_12_** and **SiO_2_imiSO_3_-PW_12_** samples at two different enlargements.

The SEM micrographs shown in Figure 8 are useful for studying the morphology of the samples investigated. In particular, Figure 8 shows the SEM images of the two samples functionalized with PW_12_ at two different magnifications. From the observation of the photos relating to the SiO_2_imi-PW_12_ sample, their morphology is identical to that of the support, whose photos are not reported for the sake of brevity. This fact indicates that the heteropolyanion is well dispersed on the surface of the support. Furthermore, as highlighted by the EDX analysis, the amount of PW_12_ is much lower than that present on the surface of the other sample (**SiO_2_imiSO_3_-PW_12_**), and this fact is also in agreement with the results obtained from the TGA curves that indicate a lower amount of PW_12_ deposited on the SiO_2_imi support compared to that deposited on the SiO_2_imiSO_3_, which is about 2.5 times less. Interestingly, the same ratio between the amounts of PW_12_ deposited on the two supports of about 1/2.5 was observed by the EDX investigation. The SEM images of the SiO_2_imiSO_3_-PW_12_ sample, as reported in Figure 8, show that, unlike the other sample, it exhibits good coverage of the support surface with PW_12_. In fact, it is possible to see only small portions of the support not covered by PW_12_. This fact can justify the significant reduction in surface area from 37 to 3.2 m^2^∙g^−1^ between the pure support and the sample functionalized with PW_12_, and, as previously hypothesized, the partial blocking of the support pores by PW_12_.

As explained above, the dehydration of hexoses is catalyzed in an acidic medium; consequently, the acidity of the catalyst’s surface is a key parameter influencing the activity of solids. The acidity of calcined materials is often determined by the temperature-programmed desorption of ammonia (NH_3_-TPD) [27]. As the materials prepared and tested in this work were not calcined, the acidity measurements were performed following the alternative method of Gervasini et al., who proposed the catalytic dehydration of 2-propanol as a test reaction to study the acid–base characteristics of sites for oxides used as catalysts [17]. The dehydration of 2-propanol to form propylene is catalyzed by acidic sites, and this reaction is considered a good measure of the overall acidic properties of the catalyst’s surface [28]. Catalytic 2-propanol dehydration, forming propene, has already been used to compare the acidity among a set of supported Keggin heteropolyacids H_3_PW_12_O_40_ on different substrates [29]. Preliminary experiments indicated that after an equilibration time (approximately 30 min) at room temperature in dark conditions, 2-propanol was partially adsorbed on the surface of all the catalysts used in this work; when the adsorption/desorption equilibrium was reached, the catalytic experiments started. Previous experiments allowed us to ascertain the optimal conditions for the catalytic reaction; consequently, the reaction lasted 2 h at a temperature of 120 °C. Reaction products were analyzed in a batch reactor at intervals of 30 min to ascertain the evolution of the batch reaction.

As studied by Misono et al., heteropolyacids possess the peculiar ability to maintain the pseudo-liquid phase inside their bulk, blocked inside the spaces between Keggin anion cluster units [30]. These spaces, referred to as secondary structures of heteropolyacids, contain acidic Brønsted sites, which are the active sites for the catalytic reactions. Polar molecules, such as alcohols, like 2-propanol, are very soluble in the pseudo-liquid phase, and consequently, the dehydration reaction proceeds to provide propylene [28,29]. Indeed, this molecule does not remain absorbed in the pseudo-liquid phase. As previously observed, propylene appeared along with traces of propanone and diisopropyl ether, whose formation was not strictly related to the Brønsted acidity [17,28,29,31]. The amount of propene was measured in the atmosphere of the reactor after the catalytic 2-propanol dehydration reaction in the presence of 0.1 g of the studied catalysts, following the order **SiO_2_imi-PW_12_** < **SiO_2_imiSO_3_-PW_12_**, where the amount of propylene measured was 1.80 × 10^−3^ < 3.32 × 10^−2^ mM, respectively. The higher amount of propene measured in the presence of **SiO_2_imiSO_3_-PW_12_** was in agreement with the higher amount of PW_12_ deposited (see TGA, EDX, and FTIR studies) onto the surface of this catalyst with respect to that present in the **SiO_2_imi-PW_12_** material.

### 2.2. Catalytic Hexoses (Fructose/Glucose) Dehydration Activity

**Figure 9:**
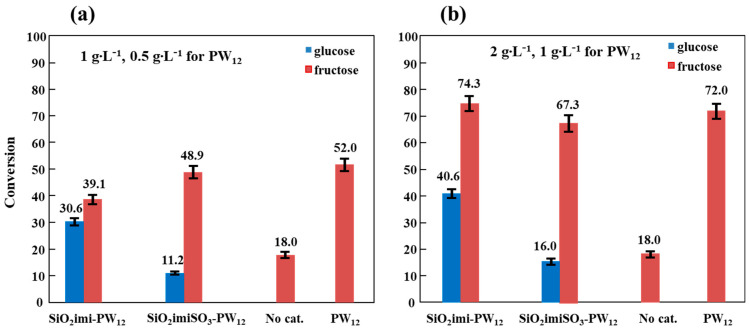
Conversion of (**a**) 1 g L^−1^ (0.5 g L^−1^ for PW_12_) glucose and fructose; (**b**) 2 g L^−1^ (1.0 g L^−1^ for PW_12_) glucose and fructose for catalytic reactions carried out in aqueous medium at 165 °C for 3 h in the presence of **SiO_2_imi-PW_12_** and **SiO_2_imiSO_3_-PW_12_,** in the absence of a catalyst, or in the presence of bare PW_12_. In the last two cases, only the dehydration of fructose was studied. The initial glucose or fructose concentration is 1 M at its natural pH.

Figure 9 reports the conversion of the two sugars obtained in the tests carried out at 165 °C for a time of 3 h in the presence of pure PW_12_ and of the two supported catalysts with two different dosages and in the absence of a catalyst. From the examination of Figure 9, it can be seen that the conversion of sugars increases by increasing the amount of catalyst used. Furthermore, pure PW_12_ has a notable capacity to convert fructose, which is generally higher than that shown by the two supported catalysts. A small quantity of fructose is instead converted in the absence of a catalyst.

With regards to fructose conversion in the presence of two supported materials, it was observed that with the lowest dosage (1 g∙L^−1^), the most active catalyst was **SiO_2_imiSO_3_-PW_12_,** while with the highest dosage, the most active catalyst was **SiO_2_imi-PW_12_** even if, considering the oscillation of the experimental value, the activity of the two catalysts at the highest dosage was similar and close to that of pure PW_12_.

This fact suggests that above a certain amount of PW_12_ present in the reactor, the activity remains constant. Instead, as far as the conversion of glucose is concerned, the activity of the two supported materials was found to be lower than that observed for the conversion of fructose, and the most performant catalyst was always **SiO_2_imi-PW_12_**. Since this catalyst, among the two supported materials, has the lower content of PW_12_ (see TGA and SEM/EDX results), it is reasonable to think that the support also contributes to the conversion of glucose, probably favoring the cross-condensation products (see Figure 1). On the contrary, catalytic tests carried out using the two pure supports (**SiO_2_imi** and **SiO_2_imiSO_3_**), results not shown in Figure 9, highlighted that the conversion of fructose is comparable to that obtained in the absence of catalyst, indicating that the catalytic activity of the two supported materials is essentially linked to the presence of PW_12_.

**Figure 10:**
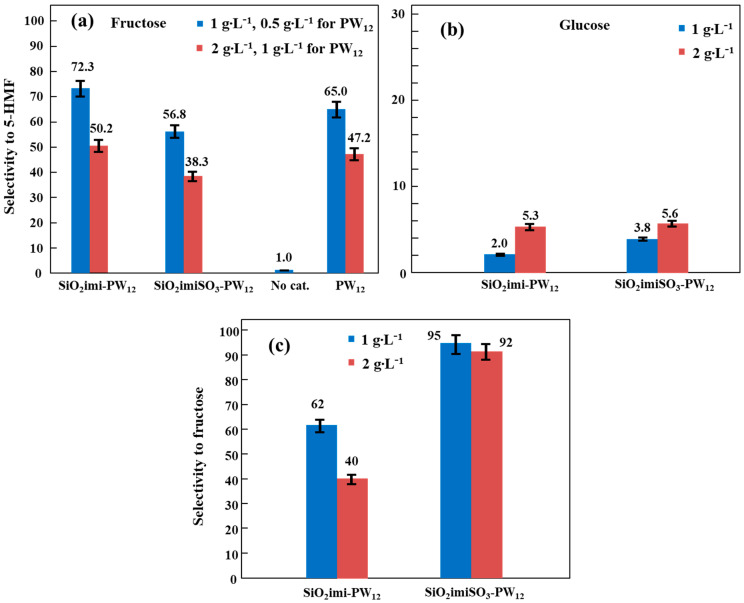
Selectivity towards the formation of 5-HMF for the catalytic test starting from (**a**) fructose or (**b**) glucose water solution (1 M), and (**c**) selectivity towards the formation of fructose for the catalytic test starting from glucose water solution (1 M). Runs carried out for 3 h at 165 °C with an amount of catalyst of 1 or 2 g∙L^−1^ in the case of **SiO_2_imi-PW_12_** and **SiO_2_imiSO_3_-PW_12_**, and 0.5 or 1 g∙L^−1^ in the case of bare **PW_12_**.

Figure 10 reports the selectivity results towards the formation of 5-HMF for the catalytic tests carried out with fructose solutions (panel a) and those carried out with glucose solutions (panel b), and in the latter case, the selectivity results towards the formation of fructose are also reported (panel c). As far as the selectivity towards 5-HMF starting from fructose solutions (Figure 10a) is concerned, it is noted that it decreases with increasing catalyst dosage, and this fact is predictable and in line with previous results. In fact, more catalyst means a greater number of acid sites and, consequently, a greater incidence of undesirable reactions, such as the rehydration of 5-HMF and, especially, the formation of humins that reduce the selectivity to 5-HMF. This fact also justifies the lower selectivity observed in the presence of the **SiO_2_imiSO_3_-PW_12_** sample compared to that observed in the presence of the **SiO_2_imi-PW_12_** catalyst, which, as highlighted by both the TGA and the SEM/EDX measurements, contains less PW_12_ and is therefore less acidic, as also observed by the acidity measurements reported in the previous characterization results section. From the study of Figure 10a, it can be observed that the selectivity towards 5-HMF in the presence of pure PW_12_ is intermediate to those obtained in the presence of the two supported catalysts. This fact can be justified by considering that the amount of PW_12_ used in the catalytic tests is intermediate to that present in the two supported catalysts. Instead, for the runs carried out in the absence of a catalyst, the selectivity to 5-HMF is very low, indicating that in this condition, the small amount of fructose reacted, as seen in the conversion reported in Figure 9, yields essentially polycondensation products.

Regarding the selectivity of 5-HMF observed in the presence of the two supported catalysts starting from glucose solutions (Figure 10b), it is possible to observe that the selectivity is very low and slightly better with a higher dosage of catalyst. This fact can be justified by considering that glucose is not directly dehydrated to 5-HMF, but it must first be isomerized to fructose. Consequently, in Figure 10c, the selectivity towards fructose obtained using the two supported catalysts is reported. From the study of Figure 10c, it is possible to see that the catalyst containing more acid sites (SiO_2_imiSO_3_-PW_12_) has a selectivity to fructose close to 100%, while the other catalyst stops at about 60% or 40%, depending on the amount used. The divergent catalytic behavior between SiO_2_imi-PW_12_ and SiO_2_imiSO_3_-PW_12_ can be justified by taking into account the different results obtained from the XPS study, namely W4f high-resolution spectra (Figure 6e,h). More specifically, SiO_2_imiSO_3_-PW_12_ shows a significant reduction degree of W atoms (40:60 W^6+^/W^5+^ atomic ratio), whereas no reduction was revealed in the case of SiO_2_imi-PW_12_. It is plausible that these findings could influence and differentiate the Brønsted/Lewis acidity balance of the two catalysts, directing their selectivity trends [32]. In addition, another noteworthy difference between the two catalysts is the higher amount of surface silanol groups present in the case of SiO_2_imiSO_3_-PW_12_ (T^2^ and Q^3^ signals; Figure 7b), which can interact with glucose, promoting its isomerization to fructose [33].

However, we can affirm that less acidic catalyst yields a higher conversion of glucose but with lower selectivity towards both 5-HMF and fructose than those observed in the presence of a more acidic catalyst. To try to explain this behavior, we must also consider the yields of 5-HMF and fructose obtained in these tests. The values of these yields are reported in Table 2, together with the conversion and selectivity data for both 5-HMF and fructose.

The data reported in Table 2 show that although in the presence of SiO_2_imi-PW_12_ the glucose conversion is much higher (approximately three times) than that observed in the presence of SiO_2_imiSO_3_-PW_12_, the yields towards the formation of 5-HMF are similar between the two catalysts. On the contrary, the yields of fructose are higher in the presence of the SiO_2_imi-PW_12_ catalyst, at least with a lower dosage, because with the higher one, the results are almost identical. This fact indicates that less acidic catalyst, despite being more able to convert glucose, essentially leads to the formation of a greater quantity of cross-condensation products (see Figure 1), resulting in a low yield of 5-HMF. The higher incidence of these unwanted reactions can be justified, as previously hypothesized, by admitting a contribution from the nanosilica support of the SiO_2_imi-PW_12_ catalyst, which, as highlighted by the SEM investigation, appears to not be completely covered by PW_12_.

To study the stability of the catalysts, three consecutive material reuse tests were conducted using both catalysts, **SiO_2_imi-PW_12_** and **SiO_2_imiSO_3_-PW_12_** (1 g·L^−1^), for the fructose dehydration reaction. These experiments demonstrated the substantial stability of these two catalysts, at least after the three consecutive reuse tests. Indeed, only a small decrease in the yield of 5-HMF was observed. In particular, the yield of 5-HMF was 28.3% in the first test, 28.0% in the second one, and 27.8% in the third cycle when **SiO_2_imi-PW_12_** was used as a catalyst, and 27.8%, 27.5%, and 27.0% when **SiO_2_imiSO_3_-PW_12_** was used as a catalyst. However, these small decreases in the yield fall within the experimental error and are due to the inevitable loss of part of the catalyst during the separation and cleaning phases between one catalytic test and the next, since after washing the catalyst, the same amount of fresh aqueous solution of sugar was added to each cycle.

### 2.3. Catalytic Treatment of Wastewater Deriving from the Brewing Industry

This section reports the results obtained from the catalytic tests carried out on wastewater from beer production. As already reported in the Experiment section, these waters contain maltose/sucrose, glucose, and fructose, and they are therefore potentially interesting for the transformation of these sugars into 5-HMF. Before proceeding with the catalytic tests, enzymatic treatment was carried out in order to transform the maltose into glucose. Table 3 reports the concentrations of the three carbohydrates before and after the enzymatic treatment.

As can be seen from the results reported in Table 3, enzymatic treatment is able to transform maltose and sucrose into glucose and fructose.

Table 4 reports the results of catalytic tests carried out on the wastewater from beer processing after enzymatic treatment in the presence of the two PW_12_-supported catalysts and bare PW_12_.

As shown in Table 4, the overall conversion of the two sugars (glucose and fructose) present in wastewater from beer processing after enzymatic treatment was higher in the presence of the **SiO_2_imi-PW_12_** catalyst. This finding is in agreement with the results obtained using synthetic solutions of glucose. In fact, after enzymatic treatment, the predominant sugar present in the solution was glucose, and as reported in Figure 8, the catalyst that gives rise to the highest conversion of glucose is **SiO_2_imi-PW_12_**. However, as already seen for synthetic glucose solutions, the selectivity and yields of 5-HMF are extremely low, indicating that the type of acid sites present in these catalysts is not suitable for transforming glucose into 5-HMF but only into fructose. Furthermore, as hypothesized in the previous section, they are able to promote parasitic cross-condensation reactions of glucose/fructose with the formed 5-HMF (see Figure 1), a fact that is particularly evident in the case of **SiO_2_imi-PW_12_**.

## 3. Experimental

The following materials were used as received without any further purification: triethoxy-3-(2-imidazolin-1-yl)propylsilane (TEIPS, d = 1.005 g/mL, MW = 274.43 g/mol, ≥97%), tetraethyl orthosilicate (TEOS, d = 0.934 g/mL 99), cetyl-trymethylammonium bromide (CTAB 98%), n-heptane (d = 0.688 g/mL 99%), hydrochloric acid (37%)—Aldrich (KGaA, Darmstadt, Germany)—ethanol (d = 0.789 g/mL ≥ 99.8%)—Fluka (Gillingham, Great Britain) - ammonium hydroxide (d = 0.90 g/mL, 30%)—Carlo Erba (Cornaredo (MI), Italy)—toluene (d = 0.87 g/mL, MW = 92.14 g/mol, ≥ 99.5%), methanol (d = 0.79 g/mL, MW = 32.04 g/mol, 100%)—VWR chemicals (Fontenay-sous-Bois, France)—H_3_PW_12_O_40_∙21 H_2_O (MW = 3256 g/mol)—Merck (KGaA, Darmstadt, Germany)—1,3-Propansultone (d = 1.392 g/mL, MW = 122.14 g/mol, 99%)—TCI, (Zwijndrecht, Belgium).

### 3.1. Preparation of the Supported Heteropolyacid Catalysts

#### 3.1.1. Preparation of SiO_2_NP 

Mesoporous silica nanoparticles’ synthesis began with ammonium hydroxide (0.8 mL, 6 mmol) base-catalyzed hydrolysis and condensation of TEOS (2.7 mL, 12 mmol) in an oil-in-water emulsion at room temperature [34]. The oil phase consisted of n-heptane (15 mL, 101 mmol), with cetyltrimethylammonium bromide (CTAB; 0.5 g, 1.3 mmol) acting as the surfactant and ethanol (5 mL, 85 mmol) as the cosurfactant–cosolvent. The mixture was maintained under continuous magnetic stirring for 4 h to facilitate nanoparticle formation. In total, 1 mL of hydrochloric acid was added to halt the base-catalyzed reaction. The product was then filtered and washed with a 1:1 water–ethanol solution to remove CTAB and the byproduct ammonium chloride. Thorough purification was achieved by a Soxhlet extractor with 250 mL of methanol for 18 h, effectively removing any residual CTAB. The final drying step at 60 °C ensured 2 g of a white solid powder (yield 80%).

#### 3.1.2. Preparation of SiO_2_imiSO_3_

**SiO_2_NP** (1 g) and the ionic liquid **imiSO_3_** [35] (2 g, 5 mmol) were dispersed in 20 mL of toluene following the method described in Vitale et al.’s work [36]. The dispersion was maintained under magnetic stirring at reflux in an argon atmosphere for 72 h. The functionalized nanoparticles were recovered through vacuum filtration on a 47-mm Omnipore™ membrane with a 0.45-μm PTFE filter. The sample was washed with toluene and then with acetone to remove unreacted imidazolinium salt, and finally, with diethyl ether. The obtained material, so-called **SiO_2_-ImiSO_3_**, was dried at 70 °C in an oven, resulting in a light-yellow powder (2 g).

#### 3.1.3. Preparation of SiO_2_imiSO_3_-PW_12_

The first heterogeneous catalyst, **SiO_2_imiSO_3_-PW_12_,** was synthesized by exploiting an acid–base reaction between phosphotungstic acid H_3_PW_12_O_40_ and sulfonic moieties of the supported ionic liquid (see Scheme 1a). To this end, an aqueous suspension of **SiO_2_imiSO_3_** (500 mg; 1.98 mmol·g^−1^) and phosphotungstic acid (3.6 g, 1.1 mmol) in a single-neck 25 mL flask was prepared and maintained under magnetic stirring at room temperature for 18 h. The immobilized catalyst was recovered through vacuum filtration on a 47-mm Omnipore™ membrane with a 0.45-μm PTFE filter. The sample was washed with distilled water and finally with diethyl ether. The obtained material was dried at 70 °C in an oven, resulting in a grayish powder (540 mg).

#### 3.1.4. Preparation of SiO_2_imi

The synthesis of **SiO_2_imi** NPs was performed through the co-condensation method following the method of Gottuso et al. [37,38,39]. TEOS (1.8 mL, 8 mmol) and TEIPS (1.1 mL, 4 mmol), in a molar ratio of 2:1, respectively, were added to the emulsion as silica sources. The synthesis follows the same procedure described for the synthesis of **SiO_2_NP**, maintaining a constant ratio between the silicon source and the other components of the emulsion. The obtained solid was recovered by vacuum filtration on a 47-mm Omnipore™ membrane, 0.45 μm in PTFE, and washed three times with distilled water and then ethanol. Thorough purification was achieved through continuous extraction with a Soxhlet extractor with 250 mL of methanol for 18 h, which effectively removed any residual CTAB. The final drying step at 60 °C ensured a stable product (760 mg).

#### 3.1.5. Preparation of SiO_2_imi-PW_12_

Analogously to the preparation of **SiO_2_imiSO_3_-PW_12_**, also to synthesize **SiO_2_imi-PW_12_**, an acid–base reaction between the phosphotungstic acid and the nucleophilic nitrogen of imidazoline belonging to the mesoporous siliceous feed was performed (see Scheme 1b). Distilled water was used as a solvent to obtain a dispersion of **SiO_2_imiNPs** (500 mg; 2.09 mmol∙g^−1^) and phosphotungstic acid (3.63 g, 1.1 mmol) in a single-neck 25-mL flask. The dispersion was maintained under magnetic stirring at room temperature for 4 h. Lastly, the material was recovered by vacuum filtration and washed multiple times with distilled water and a few milliliters of diethyl ether (664 mg).

### 3.2. Characterization of the Catalysts

*N_2_ adsorption and desorption isotherms* were recorded at 77 K after degassing the samples at 100 °C for 2 h in the degas station using a Quantachrome Nova 2200 Multi-Station High-Speed Gas Sorption Analyzer (SpectraLab Scientific Inc., Markham, ON, Canada). The specific surface area (S_BET_) was calculated through the BET method in a relative pressure (P/P_0_) range of 0.045 to 0.250. The BJH method was used to calculate the diameter size (w_BJH_) and pore size distribution within a range of 0.99 to 0.40 of the desorption curves [40].

Powder *X-ray diffraction* patterns (XRD) were acquired using a Philips PW 1050/39 diffractometer in a Bragg–Brentano geometry (Philips, Amsterdam, The Netherlands), which is equipped with a Cu Ka source (λ= 1.54056 Å), a Ni filter, and operates at an accelerating voltage of 40 kV with an emission current of 30 mA. Data acquisition occurred in a 2θ range of 2–60°, with a step size of 0.05° and a duration of 5 s per step. The crystalline phases of the samples were analyzed according to ICSD (Inorganic Crystal Structure Database) files.

*Thermogravimetric analysis* (TGA) data were obtained using a TGA/DSC STAR System (Mettler Toledo, Greifensee, Switzerland) under an airflow from 100 to 1000 °C, with a heating rate of 10 °C per minute. The temperature was increased and maintained at 100 °C for 30 min to remove adsorbed water.

FT-IR spectra were acquired using a Bruker (Billerica, MA, USA) Vertex 70v spectrophotometer, equipped with a platinum ATR and a diamond crystal (η = 2.4). Spectra acquisition occurred in the 4000–70 cm^−1^ range (2 cm^−1^ lateral resolution, 120 scans) and was analyzed using OPUS 7.5 (Bruker Instruments, Billerica, MA, USA) and Origin 2016 Software.

*ss-NMR* spectra were acquired using a Bruker (Billerica, MA, USA) Advance II 400 spectrometer (9.4 T), which operates at frequencies of 400.15, 161.97, 100.62, and 79.49 MHz for ^1^H, ^31^P, ^13^C, and ^29^Si nuclides, respectively. All samples were placed in 4-mm zirconia rotors equipped with Kel-F caps. ^13^C {^1^H} CP-MAS NMR spectra acquisition used a MAS rotation speed of 7 kHz at a temperature of 300 K, with a 90° pulse on ^1^H of 4.5 µs, a contact time during cross-polarization of 2 ms, a delay time of 3 s, and 400 scans. ^29^Si {^1^H} CP-MAS NMR spectra acquisition used an MAS rotation speed of 5 kHz at a temperature of 300 K, with a 90° pulse on ^1^H of 4.5 μs, a contact time during cross-polarization of 8 ms, a delay time of 5 s, and 400 scans.

X-ray photoelectron spectroscopy (XPS) analyses were performed with a VGMicrotech ESCA 3000Multilab (Thermo-Scientific, Waltham, MA, USA), equipped with a dual Mg/Al anode. The spectra were excited with an Al Ka source (1486.6 eV) run at 14 kV and 15 mA. The analyzer was operated in the constant analyzer energy (CAE) mode. For individual peak energy regions, a pass energy of 20 eV was set across the hemispheres. Survey spectra were measured at 50 eV pass energy. The sample powders were mounted on a double-sided adhesive tape. The pressure in the analysis chamber was in the range of 10–8 Torr during data collection. The constant charging of the samples was removed by referencing all the energies to the C1s set at 284.6 eV. The invariance of the peak shapes and widths at the beginning and end of the analyses ensured the absence of differential charging. Analyses of the peaks were carried out with the software provided by VG, based on a non-linear least squares fitting program using a weighted sum of Lorentzian and Gaussian component curves after background subtraction, as described by Shirley and Sherwood [41]. Atomic concentrations were calculated from peak intensity using the sensitivity factors provided with the software. The binding energy values are quoted with a precision of ±0.15 eV, and the atomic percentage with a precision of ±10%.

^31^P {^1^H} CP-MAS NMR spectra were acquired at a magic angle rotation frequency of 6 kHz, a 90° pulse duration on 1H of 5 μs, a contact time of 3 ms, a delay time of 2 s, and 1000 scans. Phosphorus chemical shifts were quoted with respect to phosphoric acid.

Standard samples of adamantane and tetramethylsilane optimized Hartman–Hahn’s conditions for ^13^C and ^29^Si nuclei, respectively. The two compounds were also used as external chemical shift references (29.5 and 38.6 ppm).

*Scanning electron microscopy* (SEM) and *energy-dispersive X-ray* analysis (EDX) were performed on specimens upon which a thin layer of gold had been evaporated using an FEI Quanta 200 ESEM microscope (Thermo-Scientific, Waltham, MA, USA), operating at 30 kV.

The catalytic dehydration reaction of isopropanol was used, according to Gervasini et al. [17], in order to measure the *acid–base* character of the two catalysts. For this purpose, the catalytic dehydration of isopropanol to propene was carried out in a cylindrical Pyrex batch reactor (25 mL) provided with a septum and containing 0.1 g of catalyst. After removing oxygen by flushing with N_2_ for ca. 0.5 h, liquid isopropanol (2 μL) was introduced and vaporized in the reactor (C_o_ = 1 mM), which was maintained at room temperature in dark conditions to achieve the adsorption equilibrium of the substrate on the catalyst’s surface. The reactor was then heated up to 120 °C in a static oven. The evolution of the products formed during the reaction was monitored by injecting the steam taken from the reactor, using a gas-tight syringe, into a Shimadzu GC-2010 (Shimadzu, Tokyo, Japan) equipped with a Phenomenex (Torrance, CA, USA) Zebron Wax-plus column and an FID.

### 3.3. Catalytic Hexoses Dehydration Experiments

A 50-mL Teflon-covered beaker placed in a stainless-steel autoclave heated to 165 °C was used as a reactor for the fructose and glucose dehydration reaction. The reactor was charged with 25 mL of aqueous solution of sugar (1 M) in the presence of an amount of catalyst of 1 or 2 g∙L^−1^ (0.5 or 1 g∙L^−1^ in the case of tests carried out in the presence of bare PW_12_ catalyst). The reaction lasted 3 h. The analysis and quantification of the compounds present in aqueous solution during the catalytic tests was carried out by injecting aliquots of it, at predetermined time intervals, into a Dionex Ultimate 3000 HPLC (Thermo-Scientific, Waltham, MA, USA) equipped with a REZEK ROA Organic acid H+ column (Phenomenex) and both a diode array and a refractive index detector. Finally, 2.5 mM of aqueous solution of H_2_SO_4_ were used as the eluent with a flow rate of 0.6 mL min^−1^. The UV spectra and retention times of the compounds formed were compared with those of standards (Sigma-Aldrich with purity of >99%). The catalytic activity of the catalysts in terms of the conversion (X) of hexose (i.e., fructose or glucose), selectivity (S) towards 5-HMF formation, and yield (Y) to 5-HMF were calculated as follows:

(1) $$X=\frac{{\left[hexose\right]}_{i}-{\left[hexose\right]}_{f}}{{\left[hexose\right]}_{i}}\cdot 100,$$

(2) $$S=\frac{\left[5-HMF\right]}{{\left[hexose\right]}_{i}-{\left[hexose\right]}_{f}}\cdot 100,$$

(3) $$Y=\frac{\left[5-HMF\right]}{{\left[hexose\right]}_{i}}\cdot 100,$$

where [hexose]_i_ and [hexose]_f_ are the initial and final (after 3 h of reaction) concentrations of fructose or glucose, respectively, and [5-HMF] is the concentration of 5-HMF at the end of the catalytic test. For each catalyst studied, the catalytic tests were repeated at least three times, and the conversion, selectivity, and yield values reported in the graphs are the average of the results obtained in these tests. Furthermore, in the graphs, the variations in the results from their average value are also reported.

Recycling studies on the catalysts were carried out over three consecutive catalytic runs at a concentration of 1 g∙L^−1^ of catalyst for the dehydration of fructose. For this purpose, at the end of the first catalytic test, the solid catalyst was separated from the suspension by decantation. Once the supernatant liquid phase was removed, the catalyst was washed twice with hot water and then used in a new catalytic test. This operation was repeated twice so that the same catalyst was subjected to three consecutive catalytic tests.

### 3.4. Catalytic Valorization of Sugars Contained in Wastewater Deriving from the Brewing Industry

Before the catalytic tests, the beer production wastewater was pre-treated in order to hydrolyze the maltose/sucrose it contained into glucose and fructose. The pre-treatment of beer production wastewater consisted of the enzymatic hydrolysis of maltose and sucrose. Briefly, 5.1 mL of beer production wastewater were mixed with 4.9 mL of water at pH 4.5 containing 50 μL of enzymes (Viscozyme, Novozyme). Enzymatic hydrolysis was maintained at 45 °C for 3 h. Then, the sample was filtered with a 0.45-µm membrane filter (CA, Millipore, Burlington, MA, USA) and the sugar content was analyzed using an HPLC, as explained later. Heterogeneous catalytic reactions were carried out in the presence of the home-prepared catalysts using the same autoclave/reactor described in Section 3.3. The reactor was charged with 5.6 mL of a pre-treated solution of beer production wastewater, 19.4 mL of water, and 25 mg of catalyst, or 12.5 mg in the case of bare PW_12_.

At the end of the reaction, the reactor was cooled down to approximately 25 °C. The reacted suspension was then filtered through a 0.45-µm membrane filter (CA, Millipore) and analyzed using an HPLC Agilent 1220 HPLC (Agilent, Santa Clara, CA, USA) equipped with a column Shodex SUGAR SP0810 (Tokyo, Japan) and a RID 1260 detector (Agilent). The eluent was ultrapure water at 600 μL min^−1^, the column temperature was 65 °C, and the RID temperature was 55 °C. The catalytic activity of the catalysts in terms of 5-HMF yield (Y), sugars conversion (X), and selectivity (S) towards 5-HMF formation was calculated as follows:

(4) $$Y=\frac{\left[5-HMF\right]}{{\left[total\;sugars\right]}_{i}}\cdot 100,$$

(5) $$X=\frac{{\left[total\;sugars\right]}_{i}-{\left[total\;sugars\right]}_{f}}{{\left[total\;sugars\right]}_{i}}\cdot 100,$$

(6) $$S=\frac{\left[5-HMF\right]}{{\left[total\;sugars\right]}_{i}-{\left[total\;sugars\right]}_{f}}\cdot 100,$$

where [5-HMF] is the concentration of this species measured at the end of each catalytic experiment, and [total sugars]_i_ and [total sugars]_f_ correspond to the concentration of these species present in the substrates measured by HPLC at the start and end of each catalytic experiment, respectively.

## 4. Conclusions

In this work, two different catalysts were prepared, characterized, and tested for the dehydration of fructose and glucose to 5-hydroxymethylfurfural (HMF) in an aqueous solution and for the valorization of sugars contained in wastewater from the brewing industry. These materials were the following: nanoSiO_2_ functionalized with imidazoline or with imidazoline and surface species −SO_3_^−^, and the same ones on which Keggin H_3_PW_12_O_40_ (PW_12_) was immobilized. The catalysts were physically and chemically characterized by N_2_ adsorption–desorption isotherms, XRD, TGA, FTIR, solid-state NMR, SEM, and acidity–basicity measurements. Catalytic reactions for sugar transformation were performed in batch conditions at 165 °C in the presence of suspended catalysts, and the sugar conversion, selectivity, and yield of 5-hydroxymethylfurfural (5-HMF) were determined. The valorization of sugars (fructose and glucose) was found to be more effective in the case of fructose, with a maximum selectivity of 5-HMF of approximately 72% in the presence of the **SiO_2_imi-PW_12_** catalyst. Interestingly, the two catalysts in which PW_12_ was immobilized showed activity similar to that observed for the naked PW_12_ (reaction in homogeneous phase), but with the advantage of easier separation at the end of the reaction by simple filtration. Furthermore, **SiO_2_imi-PW_12_** and **SiO_2_imiSO_3_-PW_12_** (1 g·L^−1^) were successfully recycled for three consecutive runs in the fructose dehydration reaction to obtain 5-HMF. Both catalysts proved to be very stable and easily reusable, as witnessed by the almost constant yields of 5-HMF obtained during the investigated catalytic runs. Unfortunately, tests devoted to the valorization of sugars contained in wastewater from the brewing industry yielded a poor result for 5-HMF, indicating that the catalysts prepared here were not totally suitable for this transformation. This was because the catalysts prepared in this work showed a low capacity to transform glucose (the most abundant sugar in the carbohydrate fraction of this biomass) into furans, probably because they promote parasitic cross-condensation reactions of glucose/fructose with the formed 5-HMF, a fact that is particularly evident in the case of SiO_2_imi-PW_12_. To reduce this inconvenience, it may be necessary to perform the reactions in a biphasic solvent; however, this will be the topic of future work. Indeed, the presence of another solvent in which 5-HMF is more soluble than in water would allow its removal from the reactant suspension, thus avoiding the occurrence of unwanted parasitic reactions with the formation of polycondensation compounds, which reduce the yield of 5-HMF.

## Figures and Tables

**Table 1:** Specific surface area (S_BET_), cumulative pore volume (V_t_), and pore size (w_BJH_) of the functionalized materials and the corresponding PW_12_ supported catalyst.

Sample	S_BET_ (m^2^∙g^−1^)	V_t_ (cm^3^∙g^−1^)	w_BJH_ (nm)
SiO_2_imiSO_3_	37(1)	0.15(8)	1.9(1)
SiO_2_imiSO_3_-PW_12_	3.2(2)	0.012(1)	2.0(1)
SiO_2_imi	3.9(2)	0.045(2)	1.8(1)
SiO_2_imi-PW_12_	15(8)	0.032(1)	1.7(1)

The error is indicated in parentheses as the last digit.

**Table 2:** Conversion of glucose (X), selectivity to 5-HMF and fructose (S), and yield of 5-HMF and fructose (Y) for the catalytic test of aqueous solution of glucose (1 M) in the presence of the two PW_12_ supported materials, carried out for 3 h at 165 °C.

Catalyst	Amount (g∙L^−1^)	X_glucose_	S_5-HMF_	S_fructose_	Y_5-HMF_	Y_fructose_
SiO_2_imi-PW_12_	1	30.6	2.0	62	0.6	19
	2	40.6	5.3	40	2.1	16
SiO_2_imiSO_3_-PW_12_	1	11.2	3.8	95	0.4	11
	2	16.0	5.6	92	0.9	15

**Table 3:** Carbohydrates present in wastewater deriving from the brewing industry and their concentrations before and after the enzymatic treatment.

	Concentration [mM]	
Carbohydrate	Before Enzymatic Treatment	After Enzymatic Treatment
Maltose/sucrose	273.4	-
Glucose	88.6	307.3
Fructose	28.3	98.3

**Table 4:** Conversion (X) of sugars (glucose and fructose), selectivity (S), and yield (Y) towards 5-HMF formation for catalytic runs carried out on the wastewater of the brewing industry after enzymatic treatment. Runs lasted 3 h at 165 °C with an amount of catalyst of 1 g∙L^−1^ in the case of **SiO_2_imi-PW_12_** and **SiO_2_imiSO_3_-PW_12_**, or 0.5 g∙L^−1^ in the case of bare **PW_12_**.

Catalyst	X	S	Y
SiO_2_imi-PW_12_	58.9	2.4	1.4
SiO_2_imiSO_3_-PW_12_	48.9	4.0	2.0
PW_12_	44.4	3.3	1.5

## Data Availability

Data are contained within the article and Supplementary Materials.

## Supplementary Materials

The following supporting information can be downloaded at: https://www.mdpi.com/article/10.3390/molecules30204097/s1, Figure S1: N_2_ adsorption/desorption isotherms of **SiO_2_ImiSO_3_** sample and relative pore distribution curve obtained by BJH method; Figure S2: N_2_ adsorption/desorption isotherms of **SiO_2_imiSO_3_-PW_12_** sample and relative pore distribution curve obtained by BJH method; Figure S3: N_2_ adsorption/desorption isotherms of **SiO_2_imi** sample and relative pore distribution curve obtained by BJH method; Figure S4: N_2_ adsorption/desorption isotherms of **SiO_2_imi-PW_12_** sample and relative pore distribution curve obtained by BJH method.
