# Supplementary material for: Keggin Heteropolyacid Immobilized on Nanosilica as a Heterogeneous Catalyst for Sugar Dehydration in an Aqueous Medium
Source: Molecules. 2025 Oct 15;30(20):4097. doi: 10.3390/molecules30204097 (PMC12566104; doi:10.3390/molecules30204097)
Supplement: Supplementary file 1 [file molecules-30-04097-s001.zip › molecules-3836805-supplementary.pdf]

SUPPORTING INFORMATION

Keggin Heteropolyacid immobilized on Nanosilica as heterogeneous catalyst for sugars dehydration in aqueous medium

Vincenzo Campisciano[1], Serena Lima[2], Giuseppe Marcì[2,*], Filippo Vitale[1], Maria Luisa Saladino[1], Francesco Giacalone[1] and Elisa I. García-López[1]

[1] Department of Biological, Chemical and Pharmaceutical Sciences and Technologies (STEBICEF) and INSTM UdR – Palermo, University of Palermo, Viale delle Scienze, Ed. 17 90128, Palermo, Italy

[2]Università di Palermo, Dipartimento di Ingegneria, Viale delle Scienze Ed. 6, 90128 Palermo, Italy

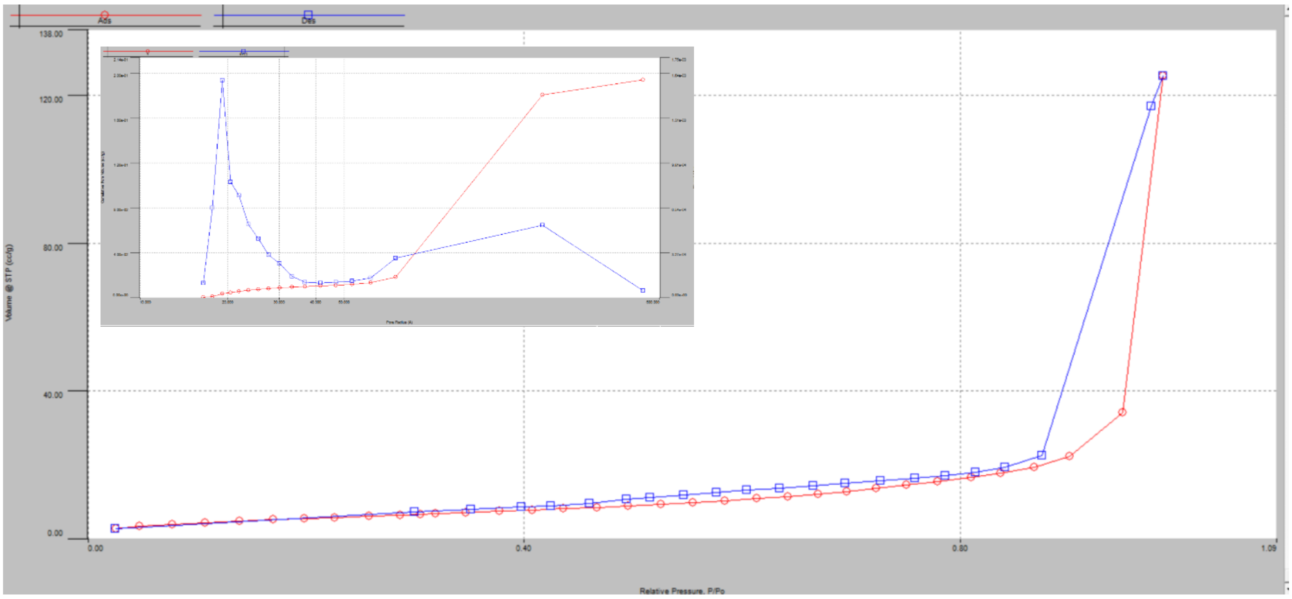

Figure S1. N_2 adsorption/desorption isotherms of **SiO_2ImiSO_3** sample and relative pore distribution curve obtained by BJH method.

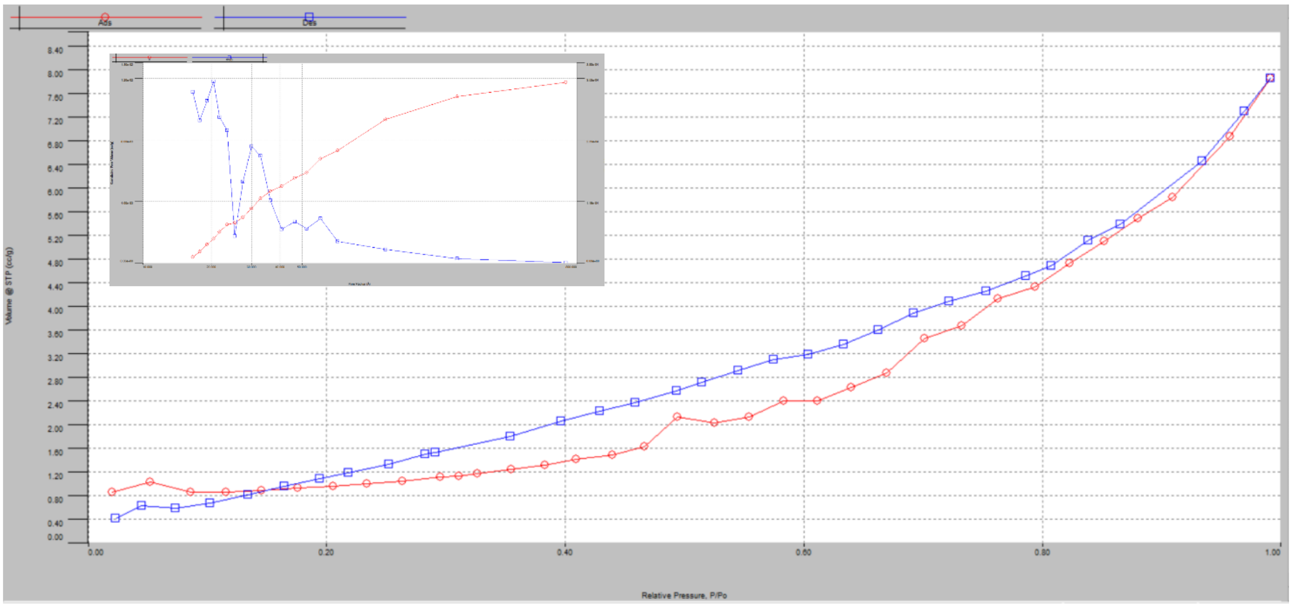

Figure S2. N_2 adsorption/desorption isotherms of **SiO_2imiSO_3-PW_{12}** sample and relative pore distribution curve obtained by BJH method.

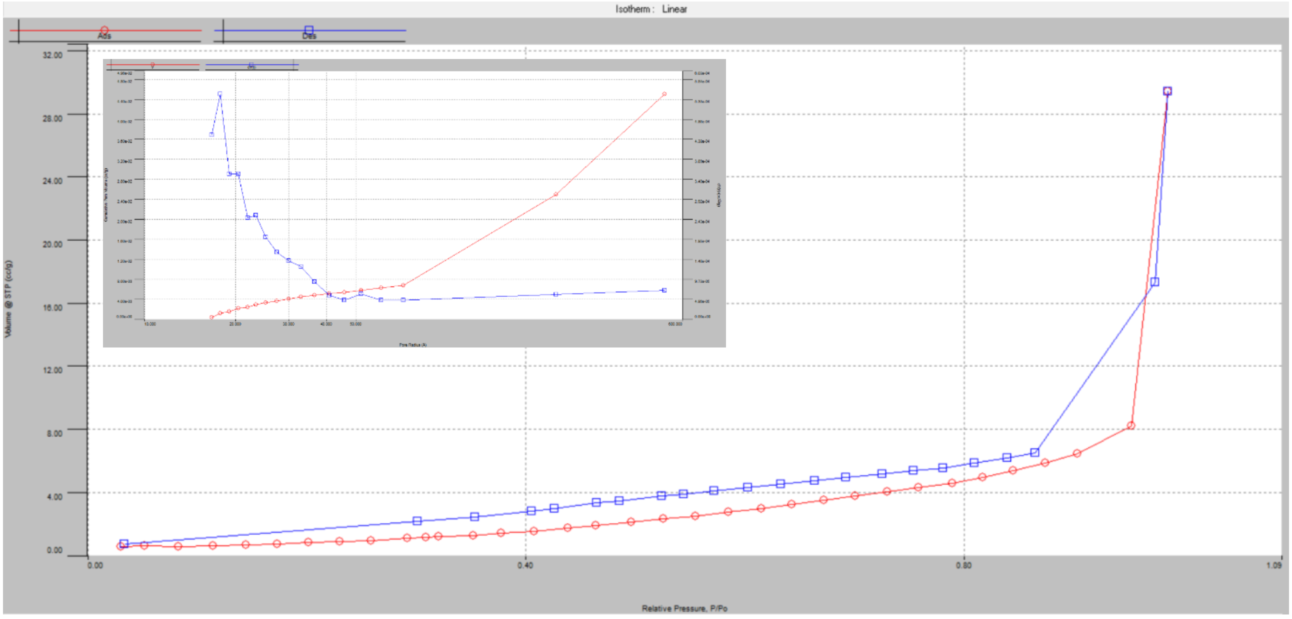

Figure S3. N_2 adsorption/desorption isotherms of **SiO_2imi** sample and relative pore distribution curve obtained by BJH method.

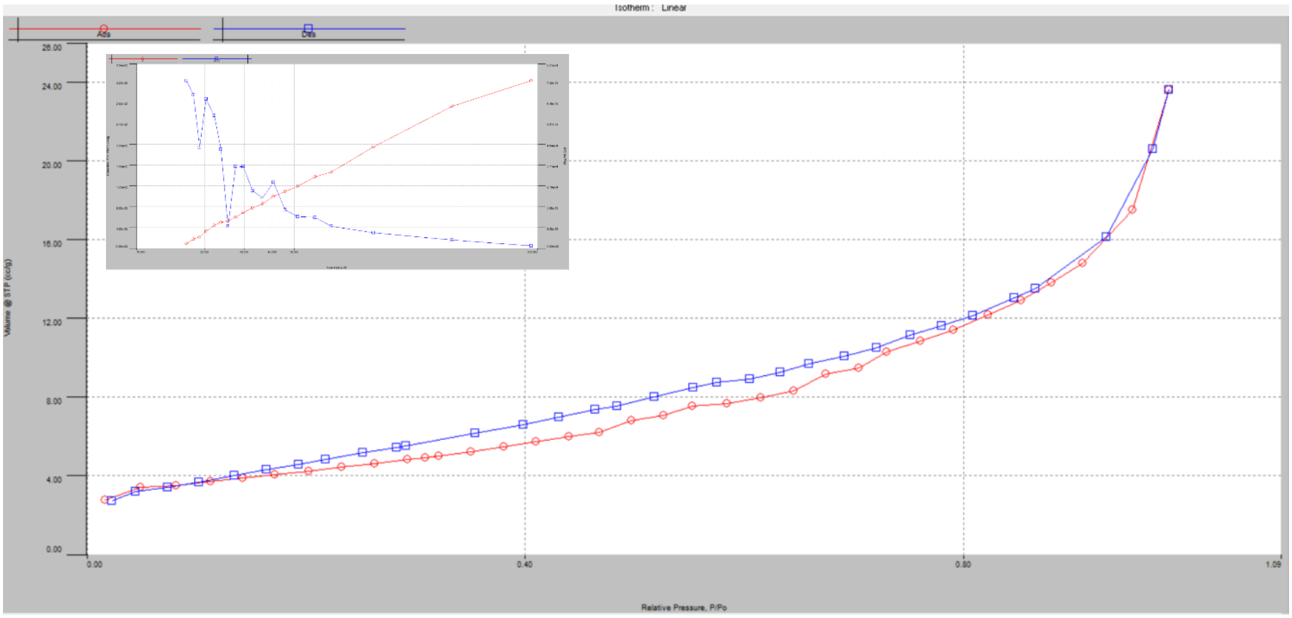

Figure S4. N_2 adsorption/desorption isotherms of **SiO_2imi-PW_{12}** sample and relative pore distribution curve obtained by BJH method.
